# Hybrid Percutaneous Intervention for a Giant Saphenous Vein Graft Pseudoaneurysm

**DOI:** 10.1016/j.jaccas.2026.108316

**Published:** 2026-05-14

**Authors:** Mina Assad Ibrahim, Christina Botrous, Nancy Z.K. Wassef, Venkatesan Suresh

**Affiliations:** a Bristol Heart Institute, University Hospitals Bristol and Weston NHS Foundation Trust, Bristol, United Kingdom; b North Bristol NHS Trust, Bristol, United Kingdom; c Gloucestershire Hospitals NHS Foundation Trust, Gloucester, United Kingdom; d University Hospitals Plymouth NHS Trust, Plymouth, United Kingdom

**Keywords:** chest pain, coronary angiography, coronary artery bypass, percutaneous coronary intervention

## Abstract

**Background:**

Saphenous vein graft (SVG) pseudoaneurysms are uncommon complications of coronary artery bypass grafting that may expand rapidly and cause compression or rupture.

**Case Summary:**

A 66-year-old man presented with non–ST-segment elevation myocardial infarction. Coronary angiography showed a patent SVG supplying the obtuse marginal branch. Six months later, he re-presented with angina, and imaging revealed a giant 80-mm SVG pseudoaneurysm compressing the pulmonary artery. Laboratory evaluation including erythrocyte sedimentation rate and C-reactive protein was negative. The patient was a surgical turndown including redo sternotomy given high operative risk. A hybrid surgical-percutaneous approach using axillary access enabled deployment of covered and drug-eluting stents, successfully excluding the pseudoaneurysm while preserving graft patency.

**Discussion:**

SVG pseudoaneurysms are rare but potentially fatal. Management depends on rupture risk, myocardial jeopardy, and surgical feasibility. Percutaneous exclusion presents a viable alternative in high-risk surgical patients.

**Take-Home Messages:**

Percutaneous exclusion of SVG pseudoaneurysms may preserve graft patency in high-risk surgical patients. Hybrid access strategies and heart team collaboration are essential in patients with complex vascular anatomy.

## Visual Summary

**Figure:**
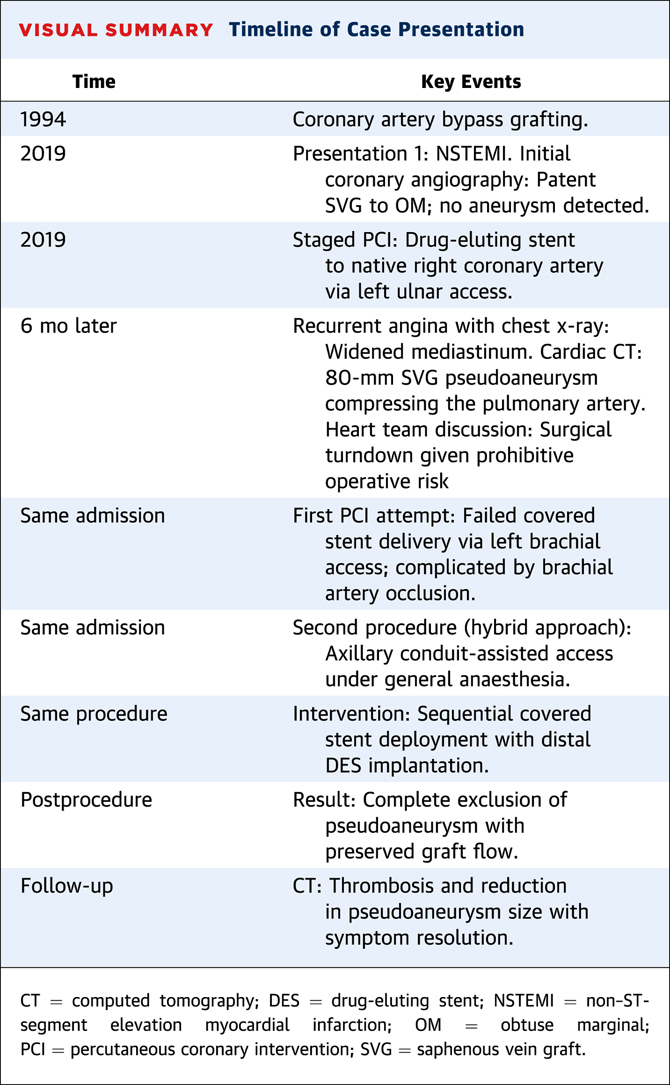

## History of Presentation

A 66-year-old man presented with non–ST-segment elevation myocardial infarction.

**Take-Home Messages**

• Saphenous vein graft pseudoaneurysms are rare but potentially life-threatening late complications of coronary artery bypass grafting.
• Percutaneous coronary intervention with covered stents may preserve myocardial perfusion when graft patency is required.
• Hybrid surgical-percutaneous access may be necessary in complex vascular anatomy.
• Multidisciplinary collaboration is critical in managing complex graft pathology.

**Figure 1:**
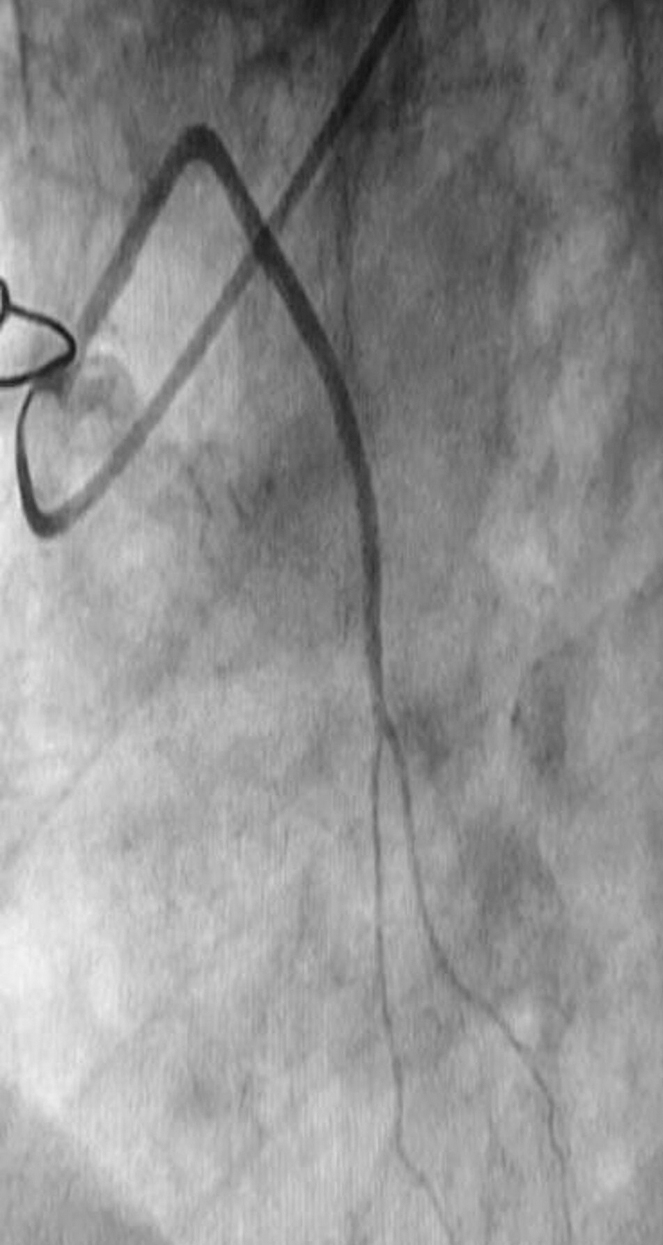
Normal Vein Graft Flow Demonstrated on the Initial Coronary Angiogram

Initial coronary angiography via left radial access demonstrated severe disease in the nongrafted right coronary artery (RCA) and a patent saphenous vein graft (SVG) supplying an occluded native obtuse marginal (OM) branch (Figure 1). A trial of percutaneous coronary intervention (PCI) to the RCA was aborted owing to severe radial spasm. A right radial access attempt was complicated by pseudoaneurysm and fistula requiring surgical repair. Subsequently, staged PCI was successfully performed via left ulnar access with drug-eluting stent implantation to the RCA.

Six months later, the patient was readmitted with further angina. Echocardiography showed preserved left ventricular systolic function. Chest x-ray showed widened mediastinum.

## Past Medical History

The patient had a history of hypertension, coronary artery bypass grafting (CABG) in 1994, abdominal aortic aneurysm repair, and peripheral arterial disease requiring femoral crossover bypass followed by right femoral endarterectomy.

## Differential Diagnosis

The differential diagnosis included acute coronary syndrome, pulmonary embolism, ascending aortic aneurysm or dissection, SVG aneurysm or pseudoaneurysm, infective graft aneurysm, mediastinal mass, and pulmonary artery aneurysm.

## Investigations

**Figure 2:**
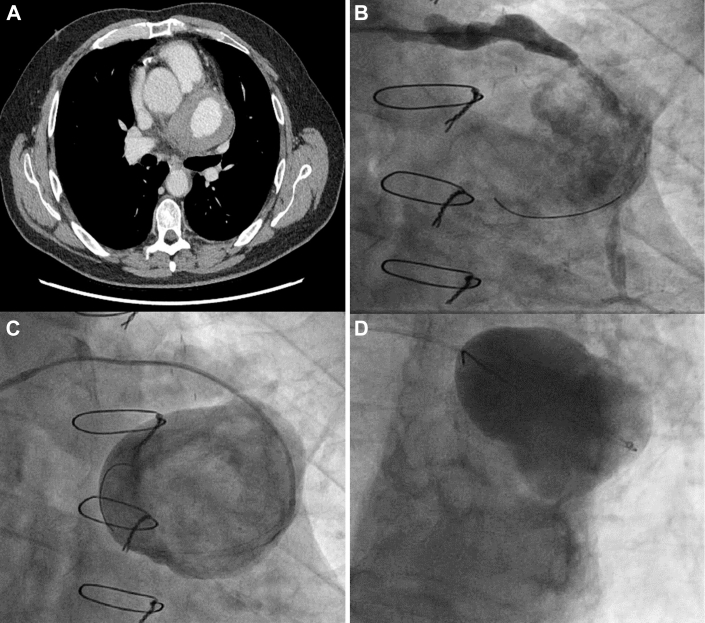
Findings on Initial Imaging Chest x-ray revealing prominence of the mediastinal contour and pulmonary artery. (A) Cardiac computed tomography showing a large pseudoaneurysm of the saphenous vein graft. (B to D) Coronary angiography showing different views of the large aneurysm.

Cardiac computed tomography (CT) revealed a giant pseudoaneurysm arising from the SVG to the OM, measuring 80 mm and compressing the pulmonary artery (Figure 2).

Right heart catheterization demonstrated normal pulmonary, right heart, and left heart pressures.

The graft had been angiographically normal 6 months earlier. Laboratory evaluation including erythrocyte sedimentation rate and C-reactive protein was negative, and there were no clinical features suggestive of infection.

## Management

A multidisciplinary discussion by the heart team concluded that redo surgery carried prohibitive risk, and the patient was a surgical turndown, including redo sternotomy considerations, owing to multiple comorbidities raising operative risk and anticipated morbidity.

Preservation of graft flow was crucial given the large myocardial territory supplied by this graft and because PCI to the native OM with chronic total occlusion was not feasible. A consensus agreement was reached for percutaneous management with covered stent implantation rather than coil embolization.

Femoral access was avoided owing to prior femoral crossover bypass, followed by right femoral endarterectomy and abdominal aortic aneurysm repair, with residual poor circulation and calcification. After multidisciplinary discussion with vascular surgeons, the femoral approach was determined to be prohibitively high risk. The vascular team advised that upper limb access was preferred to minimize the risk of infection and avoid potential damage to the existing femoral-femoral crossover prosthetic graft.

An initial PCI attempt via left brachial access ensued after failed ultrasound-guided left ulnar access. The procedure failed to deliver a 7 × 40 mm peripheral covered stent despite use of stiff wires, 8-F guide catheter, and a guide extension. The procedure was complicated by brachial artery occlusion requiring urgent vascular repair for acute left upper limb ischemia.

**Figure 3:**
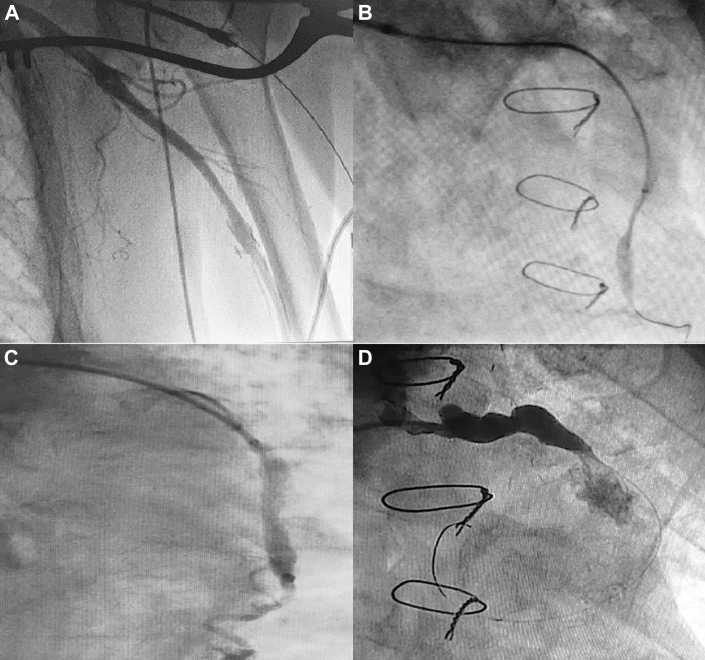
Intraprocedural Details of the Second Hybrid Procedure (A to D) Different steps of the procedure.

**Figure 4:**
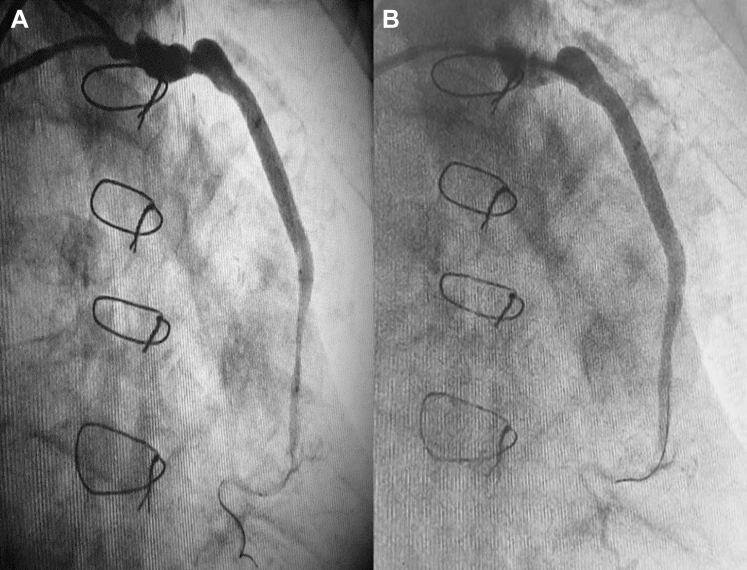
Results of the Second Hybrid Procedure (A and B) Result showing complete exclusion of the pseudoaneurysm while maintaining graft flow.

A second hybrid procedure under general anesthesia was performed with the vascular surgeon performing a left axillary cutdown with placement of an 8-F conduit sheath. This enabled delivery of a short 8-F guide catheter with a guide extension. Two covered stents were deployed, overlapping a distal drug-eluting stent, achieving complete exclusion of the pseudoaneurysm with preserved distal flow (Figures 3 and 4). The procedure was complicated by contained bleeding from an arterial branch to the pectoralis muscle, which was conservatively managed and spontaneously sealed. A summary of all complications is provided in Table 1.

**Table 1:** Complications

Event	Details
Initial coronary angiogram on presentation with NSTEMI	Right radial pseudoaneurysm with fistula that required surgical repair.
Staged successful PCI to RCA	Left ulnar artery access with no access complications.
Six months later when pseudoaneurysm was diagnosed: Aneurysm PCI initial access	Failed ulnar approach.
Failed trial to deliver covered stent	Left brachial artery occlusion after Angio-Seal required vascular surgery exploration and repair.
Successful PCI with covered stents	Left axillary cutdown complicated by contained bleeding from an arterial branch to pectoralis muscle, conservatively managed and spontaneously sealed.

NSTEMI = non–ST-segment elevation myocardial infarction; PCI = percutaneous coronary intervention; RCA = right coronary artery.

## Outcome and Follow-Up

**Figure 5:**
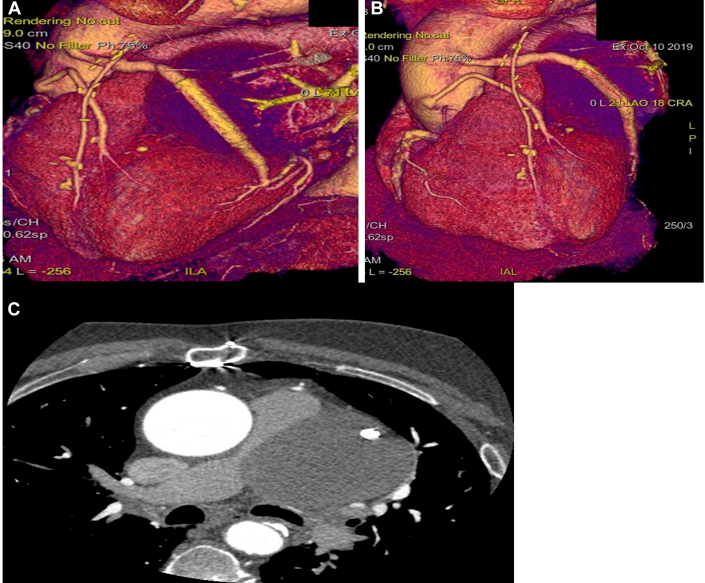
Follow-Up Computed Tomography (A to C) Postprocedural computed tomography results showing thrombosed pseudoaneurysm.

Follow-up CT confirmed thrombosis and reduction in pseudoaneurysm size (Figure 5). Clinical symptoms resolved. Isolation of the aneurysm resulted in thrombosis and shrinkage, reducing rupture risk.

## Discussion

SVG pseudoaneurysms are rare but potentially fatal complications, with a reported incidence of approximately 0.07% in large surgical series.^1^^,^^2^ Adverse event rates increase when the aneurysm diameter exceeds 40 mm.^2^ Cardiac CT is the imaging modality of choice to assess size and compression risk.

Redo CABG carries approximately 3-fold higher perioperative mortality compared with primary CABG.^3^ Percutaneous options include coil embolization or vascular occlusion devices when graft sacrifice does not compromise myocardial perfusion.^1^^,^^4^^,^^5^ Planned native coronary revascularization should be performed before graft occlusion when feasible.

In contrast to the management algorithm described by Dieter et al,^6^ native chronic total occlusion revascularization was not feasible in this case, and preservation of graft patency was essential given the large myocardial territory supplied. Therefore, covered stent implantation was selected. Hybrid conduit-assisted axillary access was required because of complex peripheral arterial disease and failure of conventional vascular routes. Multidisciplinary collaboration was central to achieving a successful outcome.

## Conclusions

SVG pseudoaneurysms are rare but carry substantial rupture risk. In high–surgical risk patients, percutaneous exclusion using covered stents represents an effective alternative to redo surgery when graft patency must be preserved. Hybrid access strategies may be required in complex vascular anatomy. Multidisciplinary heart team management is essential.

## Funding Support and Author Disclosures

The authors have reported that they have no relationships relevant to the contents of this paper to disclose.
