# Supplementary material for: Hybrid Percutaneous Intervention for a Giant Saphenous Vein Graft Pseudoaneurysm
Source: JACC Case Rep. 2026 May 14;31(24):108316. doi: 10.1016/j.jaccas.2026.108316 (PMC13309692; doi:10.1016/j.jaccas.2026.108316)
Supplement: Supplemental Figures 1 and 2 [file mmc1.docx]

A B


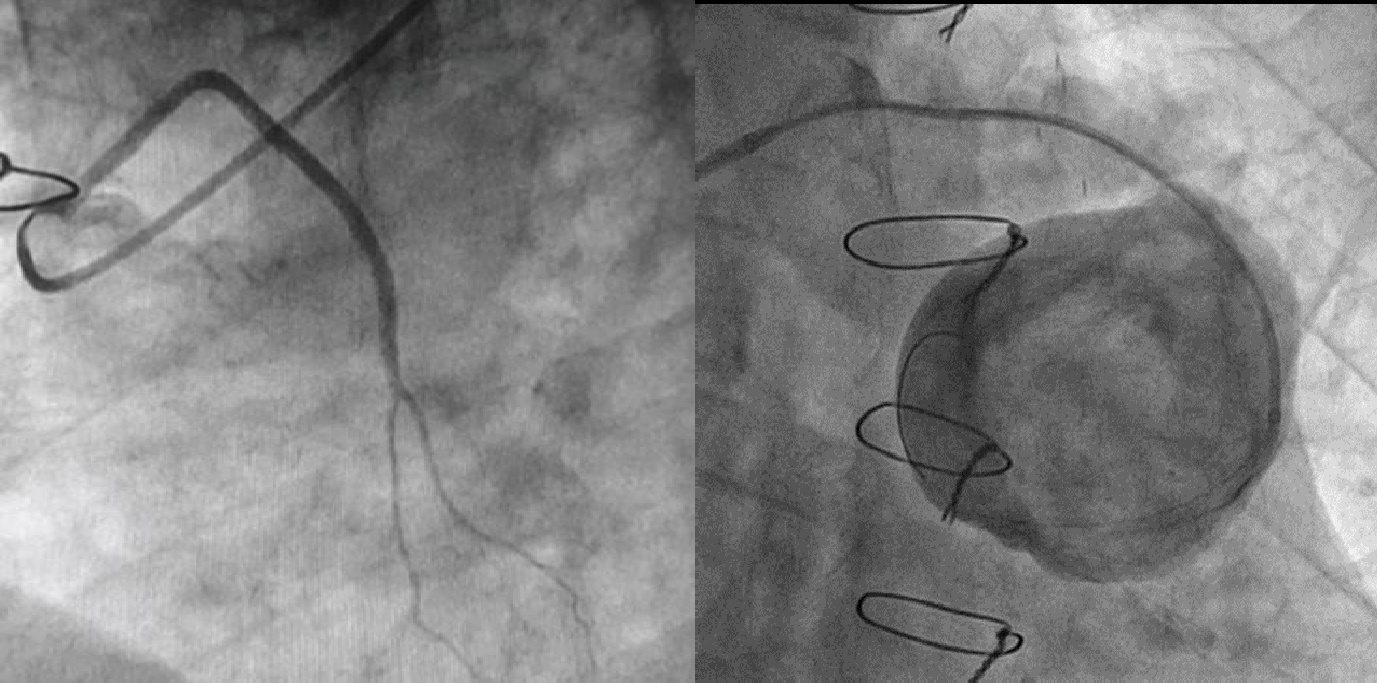

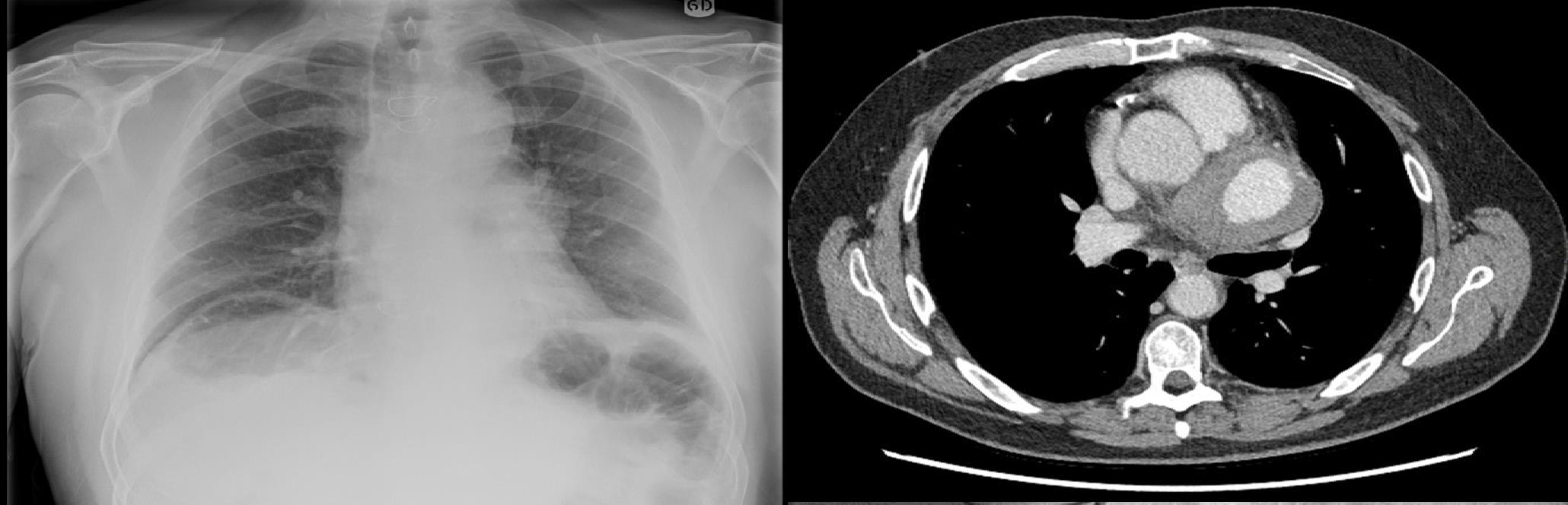


C D

# Supplementary Figure 1.

1. Coronary angiography at initial presentation demonstrating a patent saphenous vein graft without aneurysm.
2. Repeat angiography six months later showing development of a large pseudoaneurysm.
3. Chest X-ray demonstrating mediastinal widening.
4. Cardiac CT confirming a large pseudoaneurysm.

A B


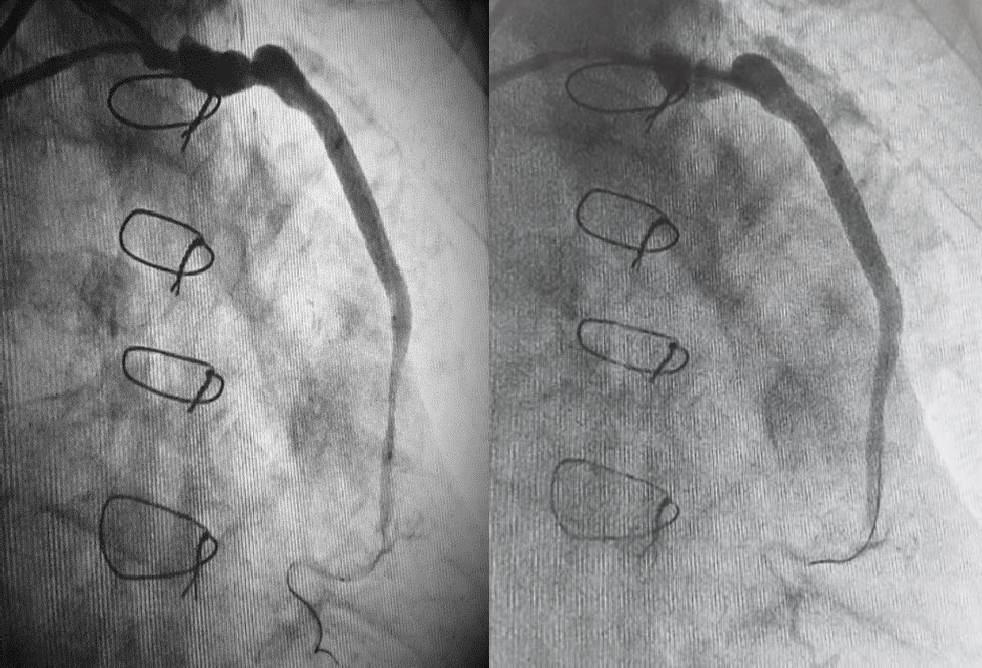

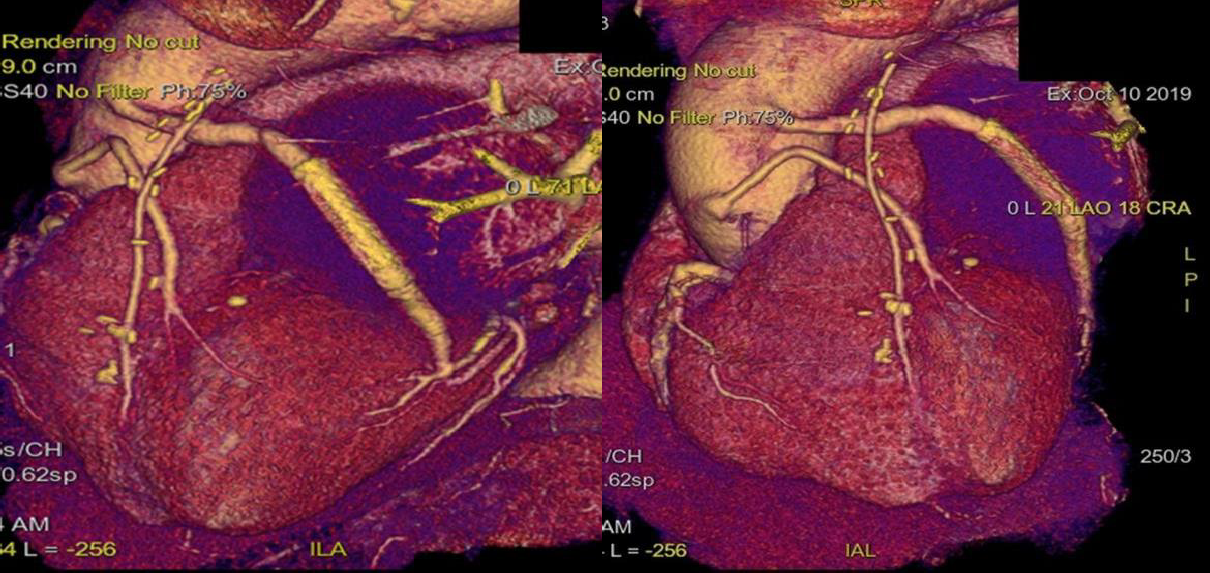


C D

# Supplementary Figure 2.

**(A, B)** Coronary angiography following covered stent implantation demonstrating exclusion of the saphenous vein graft pseudoaneurysm with preserved graft flow.

**(C, D)** Three-dimensional cardiac CT reconstructions demonstrating the covered stent within the

saphenous vein graft, confirming exclusion of the pseudoaneurysm.
